# Electrochemical Production of Hydrogen Peroxide in Perchloric Acid Supporting Electrolytes for the Synthesis of Chlorine Dioxide

**DOI:** 10.1021/acs.iecr.1c04845

**Published:** 2022-02-24

**Authors:** Mayra Kerolly Sales Monteiro, Ángela Moratalla, Cristina Sáez, Elisama Vieira Dos Santos, Manuel Andrés Rodrigo

**Affiliations:** † Institute of Chemistry, Environmental and Applied Electrochemical Laboratory, Federal University of Rio Grande do Norte, Lagoa Nova, CEP, Natal 59078-970, Rio Grande do Norte, Brazil; ‡ Department of Chemical Engineering, Faculty of Chemical Sciences & Technologies, University of Castilla-La Mancha, Campus Universitario s/n, Ciudad Real 13005, Spain

## Abstract

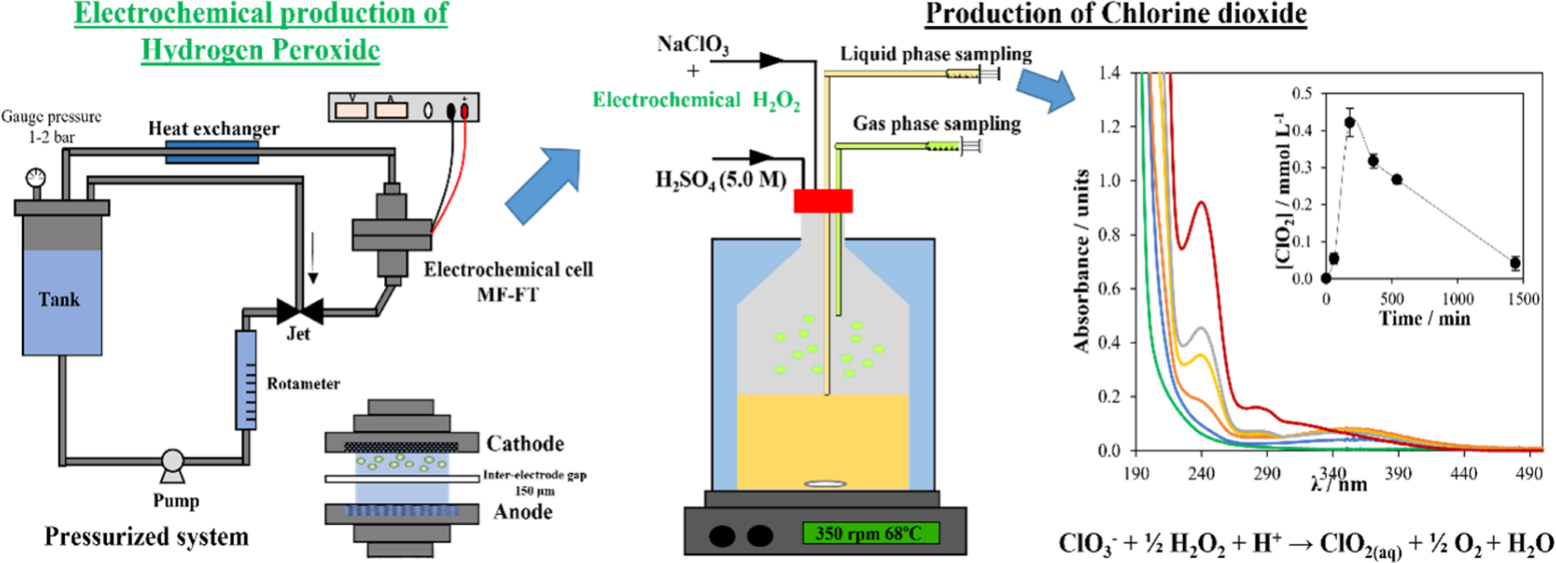

This work focuses on the electrochemical production of hydrogen peroxide in supporting electrolytes containing perchlorate ions for being used as a reagent in the reduction of chlorates to produce chlorine dioxide, as a first step in the manufacture of portable ClO_2_ production devices. This study evaluates the effect of the current density, pressure, and temperature on the production of hydrogen peroxide, and concentrations over 400 mg L^–1^ are reached. The average rate for the formation of hydrogen peroxide is 9.85 mg h^–1^, and the effect of increasing electrolyte concentration (3.0 and 30.0 g L^–1^ perchloric acid), intensity, and pressure results in values of, respectively, −2.99, −4.49, and +7.73 mg h^–1^. During the manufacturing process, hydrogen peroxide is decomposed through two mechanisms. The average destruction rate is 1.93 mg h^–1^, and the effects of the three factors results in values of, respectively, +0.07, +0.11, and −0.12 mg h^–1^. Solutions of this hydrogen peroxide produced electrochemically in a perchloric acid aqueous electrolyte were used to reduce chlorates in strongly acidic media and produce chlorine dioxide. Conversions of around 100% were obtained, demonstrating that this electrochemical product can be used efficiently to reduce chlorates to chlorine dioxide.

## 1 Introduction

In the last decade, the development of technologies capable of efficiently producing oxidizing species has been a topic of a great interest. Among the large variety of oxidants, hydrogen peroxide is capturing a great deal of attention, and its electrochemical production is now seen as one of the most promising alternatives because it has been recently found that the efficiency of the process can be increased dramatically. In addition, it may be considered as a very sustainable approach that can be easily powered with green energies.^1^

Hydrogen peroxide can be produced electrochemically via anodic and cathodic reactions.^2,3^ In the anodic route, H_2_O_2_ is produced via two-electron oxidation of water (eq 1) at the anode surface,^4^ considered as an inefficient reaction because it is difficult to stop the electrolysis for the production of hydrogen peroxide, and typically, oxygen is the final product of this anodic oxidation. The cathodic route is based on the O_2_ reduction in an aqueous medium (eq 2), a process that ends up with the formation of hydrogen, although it can be more easily promoted using electrode materials based on noble metals, noble metal alloys, or carbon.^5^ In this case, efficiencies are higher, and therefore, lower energy consumptions are required.
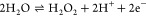
1
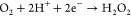
2

Several ways have been explored to enhance the production of H_2_O_2_ via this cathodic reduction, including the preparation of improved catalytic electrodes, in which the structure of the carbonaceous support is modified to stop the reduction of oxygen in hydrogen peroxide,^2,3,6−15^ or better reactor designs, based on the enhancement of O_2_ solubility by increasing the operation pressure (pressurized-jet approach), improving the contact between the electrode and catalysts by using flow-through electrodes, and reducing energy consumption by reducing the interelectrode gap (microfluidic approach).^16−20^

Thus, in a previous work, we have considered all these possibilities of improvement together, and studied the H_2_O_2_ electrogeneration in a new electrochemical reactor design based on a jet aerator and a flow-through modified carbon felt (CF) cathode using as a supporting electrolyte an aqueous solution containing 50 mM Na_2_SO_4_.^1^ As expected, it was found that the performance of the pressurized-jet microfluidic flow contributed to produce H_2_O_2_ efficiently, and the lowest energy consumption ever reported (to the knowledge of authors) was obtained (3.65 kW h kg H_2_O_2_^–1^ at 10 mA cm^–3^ in 50 mM Na_2_SO_4_).

Hydrogen peroxide has many applications in environmental remediation, but to improve its efficiency, it must be activated by its transformation into a more powerful oxidant. Thus, Fenton processes are based on the transformation of hydrogen peroxide to hydroxyl radicals, as well as other advanced oxidation processes that attain this transformation by irradiating with UV light or applying ultrasound. In the interest of transforming hydrogen peroxide into more powerful oxidants, other routes have been explored. Thus, in the last few years, H_2_O_2_ has been applied for the production of chlorine dioxide (ClO_2_) via its reaction with sodium chlorate in a concentrated acid solution such as sulfuric acid, according to eq 3.^21^ From an environmental point of view, ClO_2_ is used as an oxidizing agent for different applications such as water purification, medical treatment, sanitation, grease bleaching, aquaculture, and so forth.^22^ Also, ClO_2_ has delivered excellent performance in disinfection of drinking water because of its good properties such as no deterioration with increasing pH, which prevents the formation of chlorinated organic compounds.^23,24^

Recent research shows the promising results of this oxidant in the disinfection of personal protective equipment, besides as a solution for killing viruses such as the SARS-CoV-2 virus on surfaces or in air.^25^
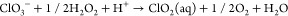
3

Chemistry of chlorine dioxide is extremely complex, and simultaneously, with the desired reactions, many other reactions can also develop, promoting the formation of chlorite, chlorine, and hypochlorous acid/chlorine in high concentrations. This highlights the need to understand the influence of the operation conditions and the ratio between hydrogen peroxide and chlorate if an efficient process is to be looked for.^26^

This work focuses on the electrochemical production of solutions rich in hydrogen peroxide using pressurized flow-through production technologies. Since the concentration of hydrogen peroxide in the process can be limited by the production of other oxidants in the cell that can react with H_2_O_2_ behaving as predators,^27^ different electrolytes have been used. The influence of temperature and pressure on the process has also been evaluated.

Finally, these solutions are prepared to be later used to produce chlorine dioxide, demonstrating the viability of the production of this oxidant with hydrogen peroxide produced electrochemically. To the authors’ knowledge, there are no other previous studies that aimed to integrate electrochemical production of hydrogen peroxide in perchloric acid supporting electrolytes and subsequent ClO_2_ production.

## 2 Materials and Methods

### 2.1 Chemicals

All the experiments were performed in a Milli-Q water solution (Millipore Milli-Q system, 18.2 MΩ cm, 25 °C). Perchloric acid (60.0% v/v) was used as a supporting electrolyte in the production of hydrogen peroxide and was supplied by Panreac. Sodium chlorate (≥99.0% w/w) was purchased from Sigma-Aldrich and used in the production of chlorine dioxide. Sodium hydroxide (4.5 N, from Hach) was used to adjust the pH. Sulfuric acid (98.0% v/v) was provided by Scharlab. Other chemicals were also analytical grade and were supplied by Scharlab. Titanium(IV) oxysulfate (1.9–2.1% v/v) was also purchased from Sigma-Aldrich and used as an indicator for hydrogen peroxide.

### 2.2 Electrochemical Production of Hydrogen Peroxide

The electrolytic tests were performed in a microfluidic flow-through cell (MF-FT) with a pressurized-jet aeration (PJA), as described elsewhere.^20^ It consisted of a tank, a pump (that supplied a constant flow rate of 140 L h^–1^), a jet aerator, a heat exchanger, and an MF-FT reactor. In this MF-FT cell, the electrodes are separated by a polytetrafluoroethylene (PTFE) film with an interelectrode gap of 150 μm. A 3D mixed metal oxide mesh (MMO-IrO_2_Ta_2_O_5_) supplied by Tianode and boron-doped diamond (BDD) supported on a 3D-niobium mesh (Diachem, supplied by Condias GmbH) were used as anodes. A 3D titanium mesh and a 3D reticulated vitreous carbon (RVC) were used as cathodic supports. These supports were modified with a mixture of carbon black (CB, Vulcan XC72 from cabot corporation) and PTFE. The modification of the cathodes was carried out following the same procedure as the one described by Moratalla et al.^20^ Perchloric acid (3000 mg L^–1^ at pH values of 1.5 and 3.0) and sulfuric acid were used as supporting electrolytes. The current intensities used in the different experiments were 0.25 and 2.50 A in the discontinuous mode without the temperature control and with the temperature control at 11.5 °C, respectively. The system worked at gauge pressures of 1.0 and 2.0 bar. A Delta Elektronika ES030-10 power supply (0–30 V, 0–10 A) provided the electric current. In order to prove that the electrogenerated hydrogen peroxide could be used for the generation of chlorine dioxide, an additional experiment was performed in the same cell using 2.7 L of the electrolyte (3000 mg L^–1^ HClO_4_) at 0.25 A, a 2.0 bar gauge pressure, and 11.5 °C in the semicontinuous mode (by feeding the electrochemical cell with 13.9 mL h^–1^ of a fresh electrolyte). The collected samples were used for the generation of chlorine dioxide. A schematic representation of the experimental setup is shown in Figure S1 in the Supporting Information.

### Production of Chlorine Dioxide

To evaluate the formation of chlorine dioxide with electrochemical hydrogen peroxide, a sample of 10 mL of H_2_O_2_ (102.3 mg L^–1^) was taken from the outlet at electrolysis (after passing 0.88 A h L^–1^) and was mixed with 1 mL of commercial sodium chlorate (32,800 mg L^–1^) and with 25 mL of H_2_SO_4_ (5.0 M) in a glass reactor (250 mL), which was completely closed. The mixture was stirred under gentle stirring conditions (350 rpm), and the temperature was set at 68 °C (with a thermostatic bath). Different samples of the liquid and gas phases were collected periodically throughout the experiment. A schematic representation of the experimental setup is shown in Figure S2 in the Supporting Information.

### Analytical Methods

Conductivity and pH were monitored using a Crison GLP31 conductivity meter and a Crison GLP22 pH meter, respectively. Hydrogen peroxide concentration in all experiments was measured using spectrophotometry following the formation of the complex between H_2_O_2_ and Ti^2+^.^28^ The chlorate concentration in the chlorine dioxide reactor was measured using a Metrohm Compact Ion Chromatograph Flex. The mobile phase consisted of 85:15 v/v 3.6 mM Na_2_CO_3_/acetone solution and was flowed at 0.8 mL min^–1^ through a Metrosep A Supp 7. The injection volume was 20.0 μL. The chlorine species in the liquid reaction mixture of the chlorine reactor were measured using an Agilent 300 Cary series UV–vis spectrophotometer. The wavelength of chlorine dioxide was found to be 360 nm. Other chlorine species as chlorite, hypochlorous acid, and chlorine were detected at 230, 323, and 303 nm, respectively.^29^ In the gas phase, two measurements were carried out. First, 5 mL of the gaseous sample was taken and bubbled in a solution containing 10 mL of water. The solution was also measured spectrophotometrically. For the second measurements, 5 mL of the gaseous sample was taken and bubbled in a solution containing 10 mL of KI (1 g L^–1^), producing the transformation from the iodide to iodine. Finally, the iodine solution was titrated with sodium thiosulfate.

## 3 Results and Discussion

### 3.1 Effect of Pressure and Current Density

**Figure 1:**
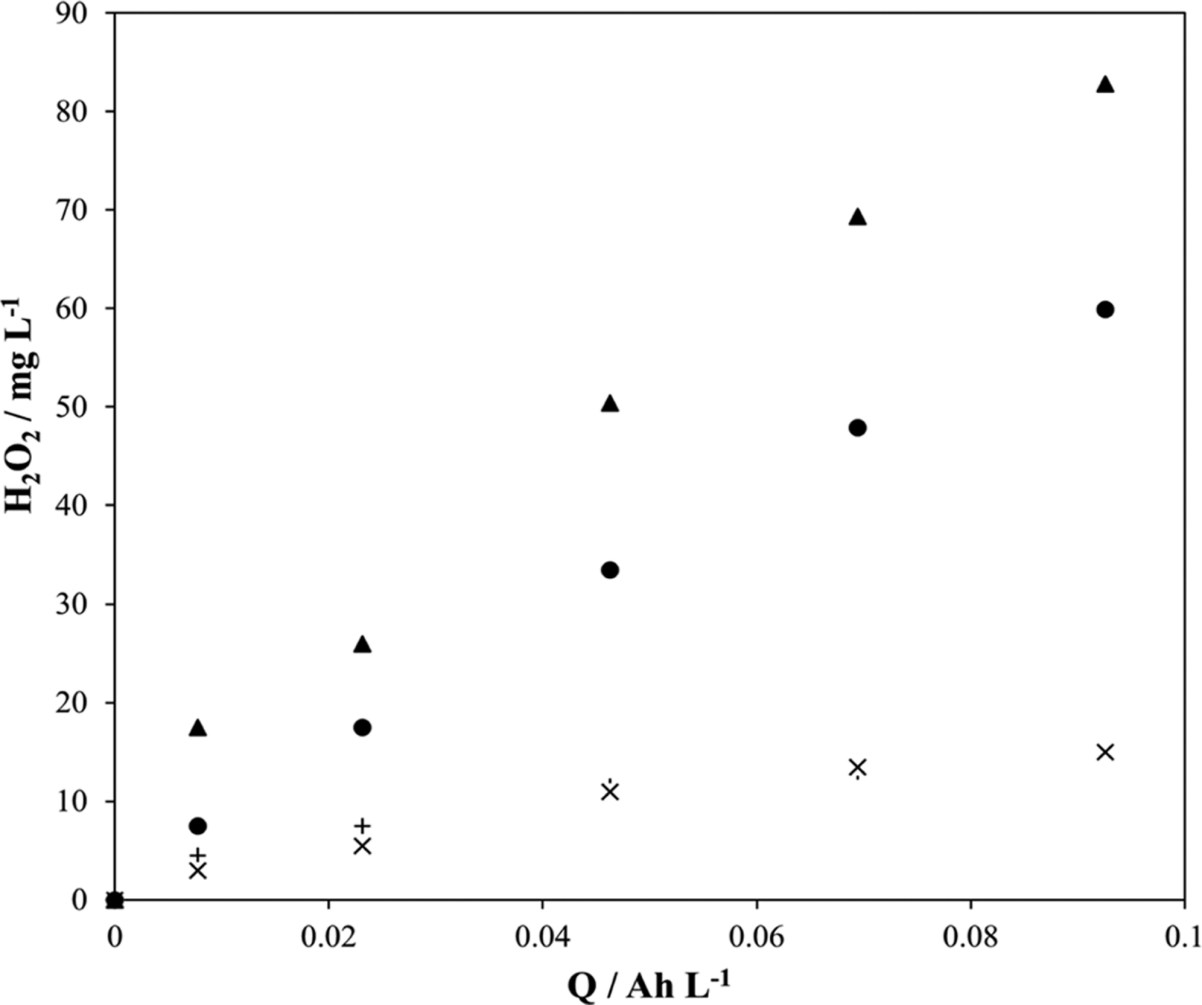
Production of hydrogen peroxide during the electrolysis of 3.0 g L^–1^ HClO_4_ at 0.25 A without regulation of temperature at 1 bar (+, *x*), at 11.5 °C, 1 bar (●), and at 11.5 °C, 2 bar (▲). Anode: MMO. Cathode: titanium mesh with CB and PTFE.

Figure 1 compares the production of hydrogen peroxide when a solution containing 3.0 g L^–1^ perchloric acid (pH = 3) is electrolyzed at 0.25 A under different pressure and temperature conditions. As seen, the operation at low temperatures and high pressures help reach higher concentrations of hydrogen peroxide, and in addition, the increase is almost linear, as expected according to the rate (eq 4), where *I* is the intensity of the current applied, η is the current efficiency, n in the number of electrons exchanged according to reaction shown in eq 2, and *F* is the Faraday constant.
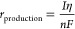
4

Results obtained are explained in terms of the higher solubility of oxygen at higher pressures and lower temperature, which contributes to minimize the diffusion controlling mechanisms of this process. Also, the decrease in the temperature may favor the decrease in the decomposition rate of hydrogen peroxide, helping in reaching higher concentrations. The test carried out without temperature regulation was carried out in duplicate in order to check the robustness of the technology replacing the electrodes. As seen, points of one run lay over the points of the other test, confirming the reproducibility of the experimental methodology applied.

**Figure 2:**
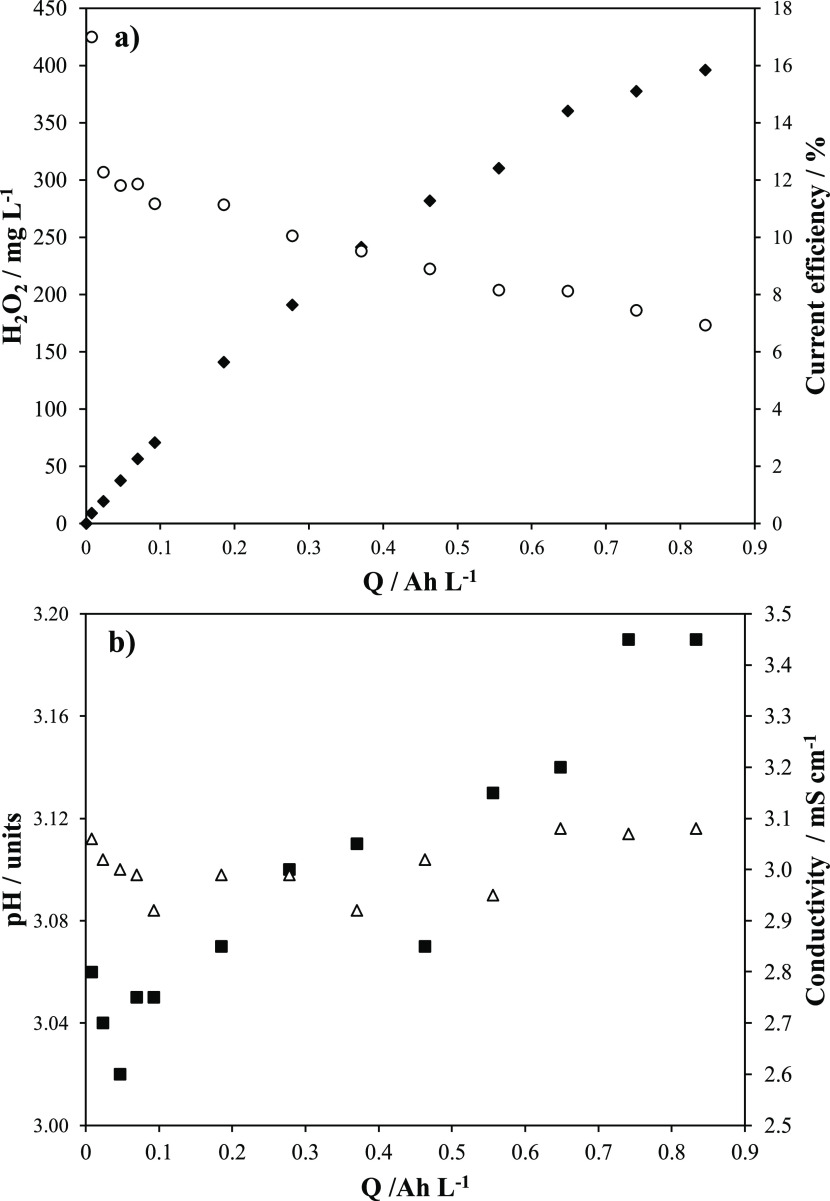
Production of hydrogen peroxide (◆), Faradaic current efficiency (◯), pH (■), and ionic conductivity (△) during the electrolysis of 3.0 g L^–1^ HClO_4_ at 0.25 A and 11.5 °C, 2 bar.

Figure 2 shows a long-term test in which a much high electric charge is passed throughout the electrochemical cell in a system operated at 2 bar and at low temperature (best conditions found in the previous tests). A very high concentration of hydrogen peroxide is reached, and the slope decays with the charge applied, which is also reflected in the decrease of the current efficiency during the electrolysis, which decays linearly from 10% (the first point seems to be outlier) down to 6%. As the supply of oxygen is continuous and the operation conditions are maintained, this decrease cannot be explained in terms of the change in the control of the rate of the process, because of the lower concentration of oxygen compared to that of other processes, as there is nothing against the transfer of oxygen flow to the electrolyte. As seen, the conductivity is maintained during the test, and the pH only increases slightly from 3.00 to 3.45, but this change cannot explain the decrease in the rate of formation of hydrogen peroxide.

Oppositely, this decrease can be explained in terms of the chemical decomposition of hydrogen peroxide, which is directly related to the concentration accumulated in the electrolyte according to eq 5
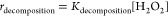
5

Hence, as the concentration of hydrogen peroxide increases, the rate of destruction increases up to the moment in which both the generation and the decomposition rate are balanced (eq. 6)
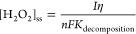
6

At this point, a steady-state concentration is reached, and the process becomes completely inefficient because all hydrogen peroxide produced is rapidly destroyed.

**Figure 3:**
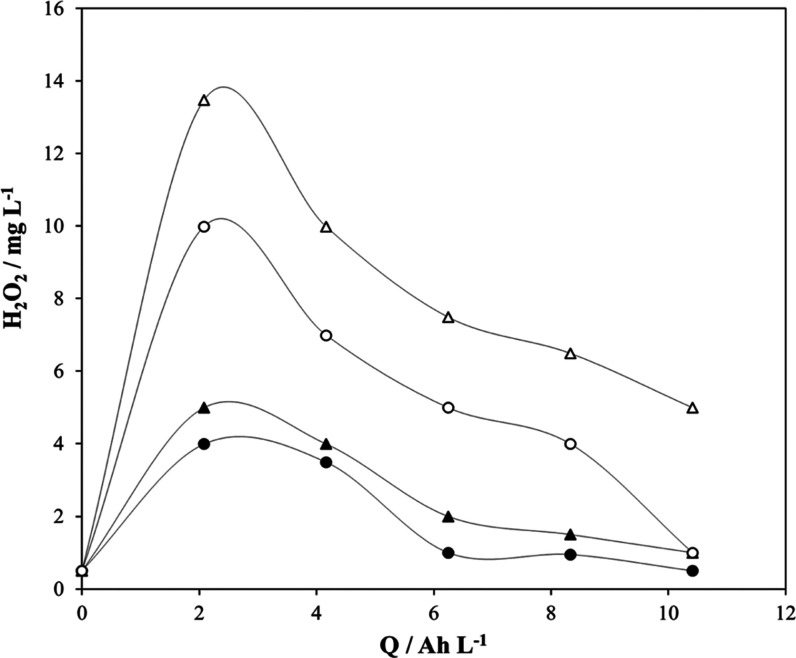
Production of hydrogen peroxide during the electrolysis of HClO_4_ at 0.25 A without temperature regulation (△) at 3.0 g L^–1^, 2 bar; (▲) 3.0 g L^–1^, 1 bar; (◯) 30.0 g L^–1^, 2 bar; and (●) 30 g L^–1^, 1 bar.

For larger specific electric charges, the situation is even worse, and a decay in the concentration of hydrogen peroxide is observed, as shown in Figure 3, in which charges 1 log more than those used in Figure 2 and 2 logs more than those used in Figure 1 are used. This decay cannot be explained if a third species is not considered as it has been recently proposed to explain the limited electrochemical production of ozone. This species is expected to react with hydrogen peroxide and destroy it. Initially, this accumulation of hydrogen peroxide predator species in the electrolyte may correspond to ozone (or any other oxidant) because when two oxidants are combined, they can be transformed into radicals that in the absence of compounds susceptible to oxidation can be later transformed into oxygen. This is what happens in the peroxone system when hydrogen peroxide and ozone are combined to form hydroxyl radicals. This system attains a very high removal rate of organics in the treatment of wastewater, but in the absence of species ready to be oxidized, the hydroxyl radicals formed are recombined, finally yielding oxygen.

As seen in Figure 3, the rate of formation of the hydrogen peroxide increases with pressure and decreases with the concentration of perchloric acid in the electrolyte. However, the rate of decrease is almost constant and does not seem to depend on these two parameters (the decay trends are almost parallel down to low concentrations of hydrogen peroxide, where the rate starts to be affected by this lower concentration). Perchloric acid was chosen because of its electrochemical inertness. Most of the anions are transformed during electrolysis into species with a higher oxidation state (e.g., sulfates into peroxosulfates, carbonates into peroxocarbonates, chlorides into chlorine, nitrates into peroxonitrates, and so on), but as perchlorate cannot produce any more oxidized peroxoanions, because it is in its highest oxidation state, this formation is not expected for it. For this reason, only species such as ozone are expected to act as predators. However, because of the very fast interaction with hydrogen peroxide, they are not expected to be detected, and what we can see is simply their effect in the increased decomposition of hydrogen peroxide.^27,30,31^ Additionally, it is important to take into consideration that oxygen solubility depends inversely on the salinity of the reaction media. Then, the higher the perchloric acid concentration is, the lower the concentration of dissolved oxygen is. This can clearly limit the electrogeneration of hydrogen peroxide.

**Figure 4:**
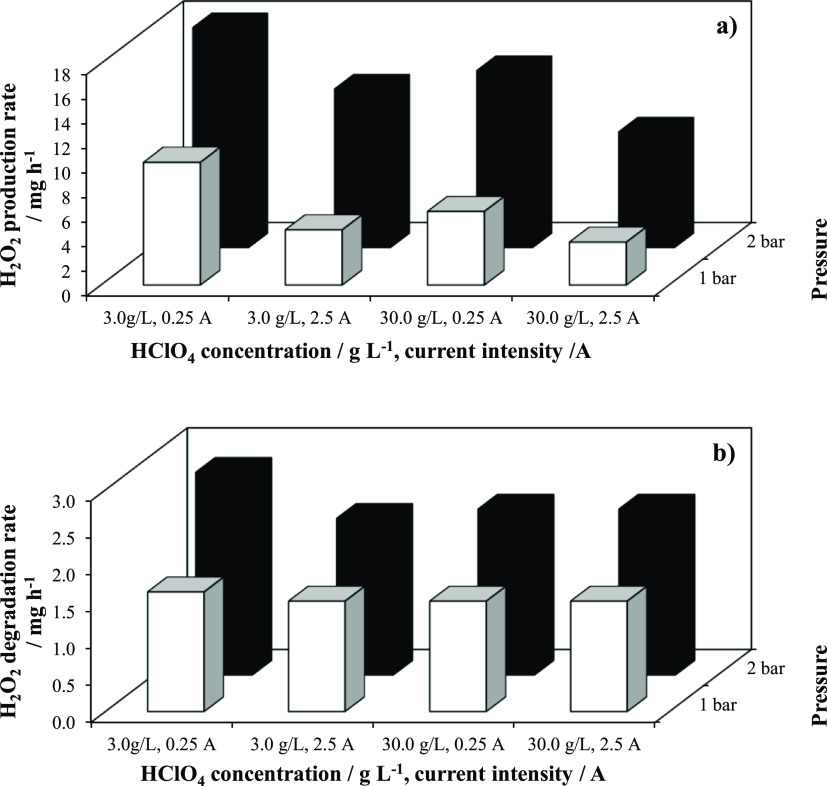
Influence of the concentration of perchloric acid, current intensity, and pressure on the formation (a) and decomposition rates (b) of hydrogen peroxide. White bars: 1 bar and black bars: 2 bar.

Figure 4 compares the influence of the concentration of perchloric acid, current intensity, and pressurization on the formation (Figure 4a) and decomposition rates (Figure 4b) of hydrogen peroxide. As seen, pressure is the most important parameter in the production of the oxidant, which is also favored when working at lower current densities and with a not highly concentrated perchloric acid solution. Decomposition rates seems to follow the same trends (although more laminated), and this can be explained by considering that these rates are directly related to the concentration reached of hydrogen peroxide because the decay rates depends directly on this concentration.

If the effects are compared among the three factors (in fact, experiments shown in this figure followed a 2^3^ design of experiments), it can be seen that the average rate for the formation of hydrogen peroxide is 9.85 mg h^–1^ and the effect of increasing concentration, intensity and pressure results in the rate changing to, respectively, −2.99, −4.49, and +7.73 mg h^–1^. Regarding the destruction rate, the average value is 1.93 mg h^–1^ and the effects of the three factors results in values of, respectively, +0.07, +0.11, and −0.12 mg h^–1^.

**Figure 5:**
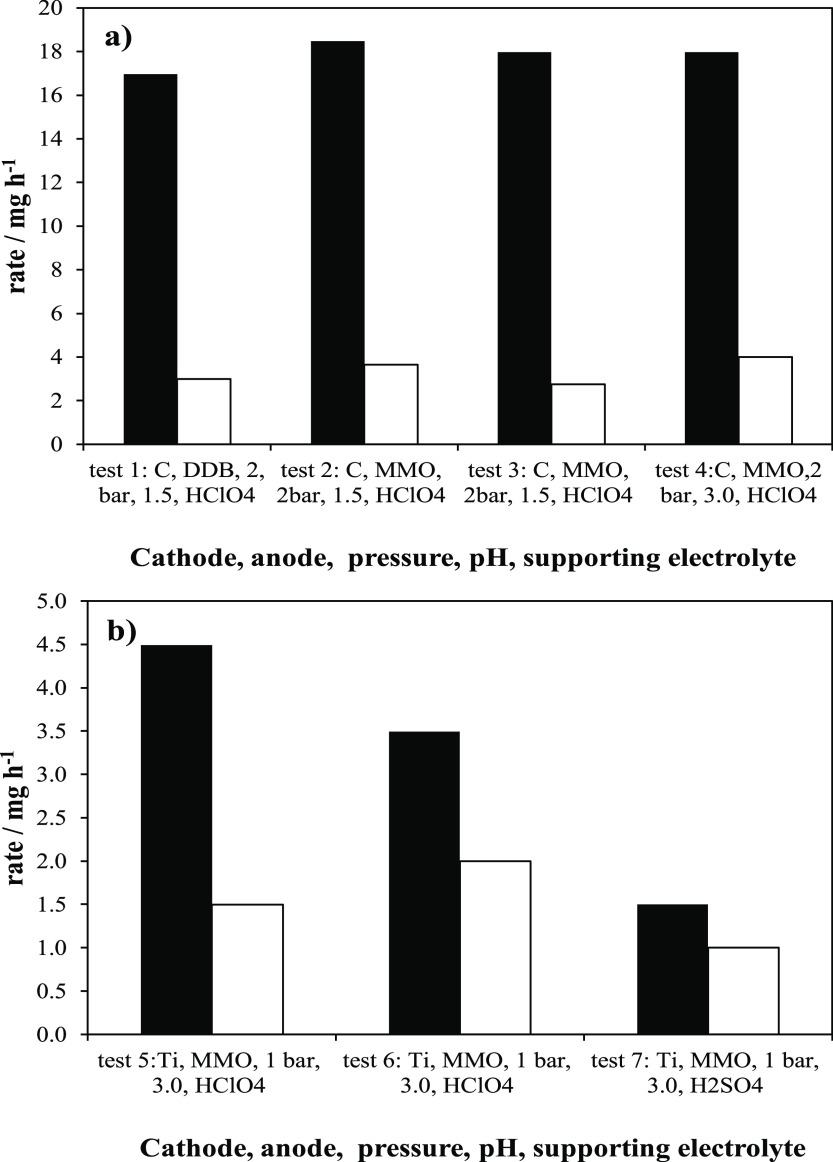
Influence of the electrode material, operation pressure, and formulation of the electrolyte in the production of hydrogen peroxide at 0.25 A. Black bars: production rate and white bars: decomposition rate.

Figure 5 summarizes the production and decay rates with different anodes (MMO and BDD), cathodic supports (titanium mesh and RVC foam), and electrolytes (HClO_4_ and H_2_SO_4_). In Figure 5a, tests carried out at 2 bar are shown. As seen, there are no relevant differences between the use of diamond BDD or mixed metal oxides MMO coatings. This is expected because of the secondary role of the anode in this process. This means that destruction of hydrogen peroxide in the anode is not promoted with the use of an electrode such as diamond, well-known for its efficiency in the production of hydroxyl radicals. The same can be stated with respect to electrolyte pH, with no relevant changes within the range 1.5–3.0, suggesting that the stability of the hydrogen peroxide produced is high under these conditions. Test 3 is a repetition of test 2 with a different cathode (although with the same composition), carried out to check again the reproducibility using different electrodes and confirming the robustness of conclusions drawn.

Regarding Figure 5b, compiling the results obtained at 1 bar, test 6 is carried out again to check the reproducibility of the cathode on a different support (titanium mesh instead of RVC foam). Again, it is confirmed. Regarding test 7, it points out significant differences in using sulfates instead of perchloric acid as the electrolyte. The second can be related to the formation of peroxosulfates, oxidants capable of interacting with hydrogen peroxide and reducing the overall efficiency of the system. Comparing Figure 5a,b, again, the pressure behaves as the key parameter to obtain high efficiencies.

### 3.2 Production of Chlorine Dioxide with Electrochemically Produced Hydrogen Peroxide

Our interest is to produce hydrogen peroxide electrochemically as an intermediate stage in the production of chlorine dioxide, which is a more powerful oxidant. It is aimed to produce large concentrations in a very simple process, and the supporting electrolyte is key in reaching this objective. With the aim to verify that the hydrogen peroxide produced electrochemically in the perchloric acid supporting electrolyte can be used to produce chlorine dioxide, a test was carried out with our experimental setup to produce hydrogen peroxide (0.25 A, 2 bar) using a perchloric acid electrolyte (3000 mg L^–1^ HClO_4_) and operated in the semicontinuous mode by feeding the cell with an average flow rate of 13.9 mL h^–1^ of fresh perchloric acid solution. The outlet flow was mixed with chlorate and sulfuric acid to produce chlorine dioxide. The steady-state concentration reached after passing 0.88 A h L^–1^ in this system was 102.3 mg L^–1^, and hydrogen peroxide is contained in an aqueous matrix with 3000 mg L^–1^ HClO_4_. A sample of 10 mL of this solution was mixed with 1 mL of commercial sodium chlorate (32,800 mg L^–1^) and with 25 mL of 5.0 M H_2_SO_4_ to evaluate the formation of chlorine dioxide.

**Figure 6:**
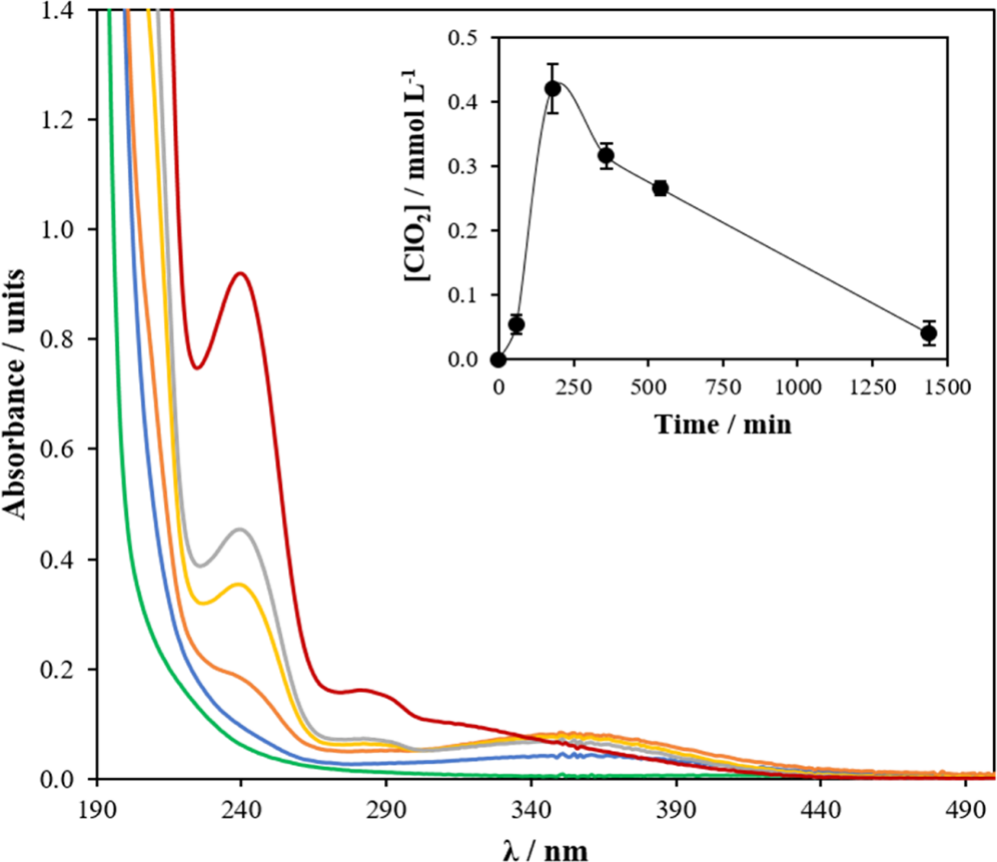
Changes in UV–vis spectra during the production of chlorine dioxide in the liquid reaction mixture. The inset panel shows the concentration of chlorine dioxide produced in the system during the reaction. The error bars represent the standard deviations from duplicate tests: Legend: green line, 0 min; blue line, 60 min; orange line, 180 min; yellow line, 360 min; gray line, 540 min; and red line, 1440 min.

Interaction between oxidants in electrochemical systems is rather complex, and measurement can significantly interfere with the interpretation of results. Because of this, it was decided to follow the formation of chlorine dioxide spectrophotometrically. Results are shown in Figure 6, where an efficient production of chlorine dioxide (peak at 360 nm) can be observed, which is also accompanied with the production of chlorite (peak 230 nm) and the pair hypochlorous acid (323 nm)/chlorine (310 nm). Concentration of chlorine dioxide increases up to 0.42 mmol L^–1^ from the very beginning and then decreases slowly because of the transformation to the gas phase and the further reduction of chlorine dioxide, while the concentrations of chlorite and hypochlorite/chlorine increases significantly over time, highlighting that chlorine dioxide is not the final product but an intermediate in the reduction of chlorate, as indicated in eq 7.
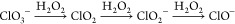
7

Production of chlorite is not negative because this anion can be easily transformed into chlorine dioxide, but it should be pointed out that the stoichiometry of the process must be seriously controlled in order to promote the formation of the desired product. The residence time in the electrochemical reaction also seems to be a very important issue to be consider. The final sample was analyzed, and it contained 710 mg L^–1^ chlorate, indicating that this species was not limiting the process. Oppositely, hydrogen peroxide was depleted. The total consumption of hydrogen peroxide was 0.88 mmol L^–1^, and the maximum concentration of chlorine dioxide produced, that is, 0.42 mmol L^–1^, was obtained after 3 h of operation . Considering the stoichiometry of the process, this means that the maximum efficiency in the production of chlorine dioxide was 95.4%. As explained before, the decay in the concentration of chlorine dioxide may be explained in terms of the further decomposition of chlorine dioxide and its stripping into the gas flow.

**Figure 7:**
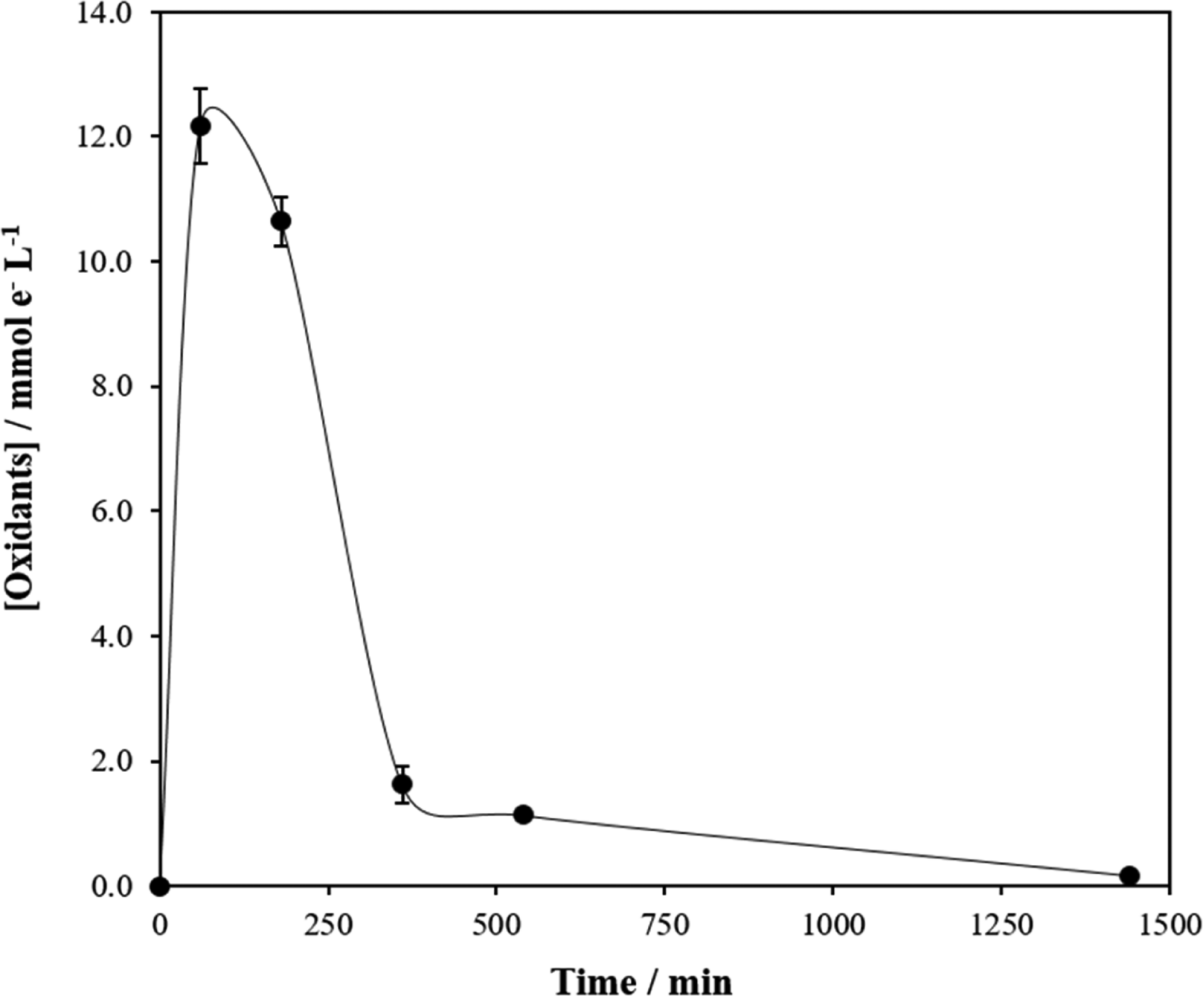
Evolution of oxidants in the gas during chlorine dioxide production. The error bars represent the standard deviations from duplicate tests.

**Figure 8:**
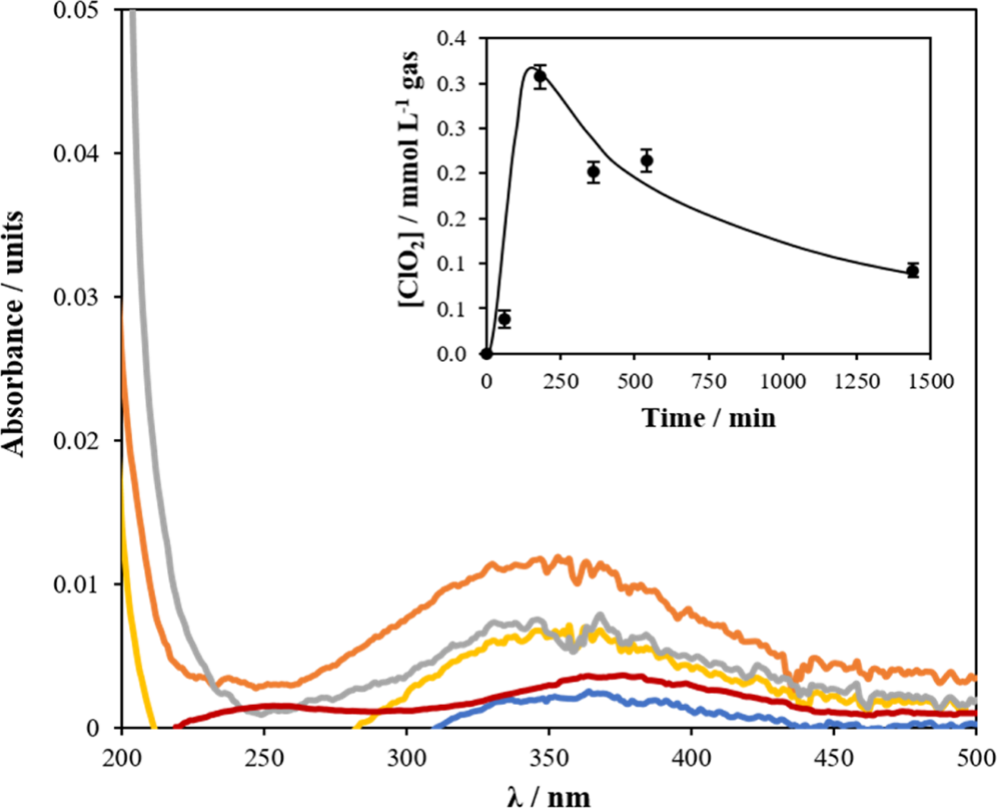
Changes in UV–vis spectra of the gas bubbled in water during the chlorine dioxide production test. The inset panel shows the concentration of chlorine dioxide produced in the system during the reaction. The error bars represent the standard deviations from duplicate tests. Legend: blue line, 60 min; orange line, 180 min; yellow line, 360 min; gray line, 540 min; and red line, 1440 min.

Thus, regarding the gas, the capacity of oxidation (measured as the concentration of iodide oxidized to iodine) is shown in Figure 7, and the UV spectra of the gas collected into water are shown in Figure 8. Regarding the concentrations of the oxidant produced, it confirms the formation of gaseous oxidant species in the reactor and that they are stripped during the experiment and can be used as a gaseous oxidant. The UV spectra of this gas shown in Figure 8 indicate that a much lower concentration of chlorine dioxide is observed and also that in this case, the peaks of chlorine and hypochlorous acid are not detected. The height of the absorption peak matches with the concentration of the oxidant measured (with a certain delay taking into account that gaseous samples were taken in the reactor and the oxidants were measured in a separated tank), and the maximum is reached at 3 h. It is important to consider that the chlorate was added only at the beginning and the concentration in the reaction tank decreases form 714 mg L^–1^ to 710 mg L^–1^, so this confirms the depletion of this reagent (semicontinuous operation mode).

Hence, the production of chlorine dioxide from the oxidation of chlorates with electrochemically produced hydrogen peroxide is feasible and leads to significant conversions in the limiting reagent.

## 4 Conclusions

This study demonstrated that hydrogen peroxide can be efficiently produced by the electrolysis in perchloric acid solutions at 0.25 A and 2 bar of gauge pressure. The average rate for the formation of hydrogen peroxide was 9.85 mg h^–1^, and the effect of increasing concentration, intensity and pressure resulted in the rates of, respectively, −2.99, −4.49, and +7.73 mg h^–1^. Regarding the destruction rate, the average value iswas 1.93 mg h^–1^, and the effects of the three factors resulted in rates of, respectively, +0.07, +0.11, and −0.12 mg h^–1^. Furthermore, it was found that electrochemically produced hydrogen peroxide (contained in a matrix of perchloric acid) can be successfully used to produce chlorine dioxide. Maximum efficiencies of around 100% were obtained; however, chlorine dioxide behaved as an intermediate, and it was transformed into other chlorinated species. The maximum concentrations of chlorine dioxide found was 0.42 mM when 10 mL of electrochemically produced hydrogen peroxide was mixed with 1 mL of commercial chlorate (32,800 mg L^–1^) and with 25 mL of H_2_SO_4_ 5.0 M. The liquid product produced also contained chlorine and chlorite, but gases were free from these species.

## Supporting Information Available

Additional experimental details including the schematic representations of the experimental setups (PDF)
