# Supplementary material for: Electrochemical Production of Hydrogen Peroxide in Perchloric Acid Supporting Electrolytes for the Synthesis of Chlorine Dioxide
Source: Ind Eng Chem Res. 2022 Feb 24;61(9):3263–71. doi: 10.1021/acs.iecr.1c04845 (PMC8919508; doi:10.1021/acs.iecr.1c04845)
Supplement: Supplementary file 1 — ie1c04845_si_001.pdf [file ie1c04845_si_001.pdf]

**Supplementary material for**

# Electrochemical production of hydrogen peroxide in perchloric acid supporting electrolytes for the synthesis of chlorine dioxide

Mayra Kerolly Sales Monteiro[1,2], Ángela Moratalla[2], Cristina Sáez[2], Elisama Vieira Dos Santos[1], Manuel Andrés Rodrigo[2,*]

[1] Institute of Chemistry, Environmental and Applied Electrochemical Laboratory, Federal University of Rio Grande do Norte, Lagoa Nova, CEP 59078-970, Natal, RN, Brazil

[2] Department of Chemical Engineering, Faculty of Chemical Sciences & Technologies, University of Castilla-La Mancha, Campus Universitario s/n, 13005 Ciudad Real, Spain

*author to whom all correspondence should be addressed (corresponding author): manuel.rodrigo@uclm.es Tel.: +34-926-29-53-00 Ext. 3411

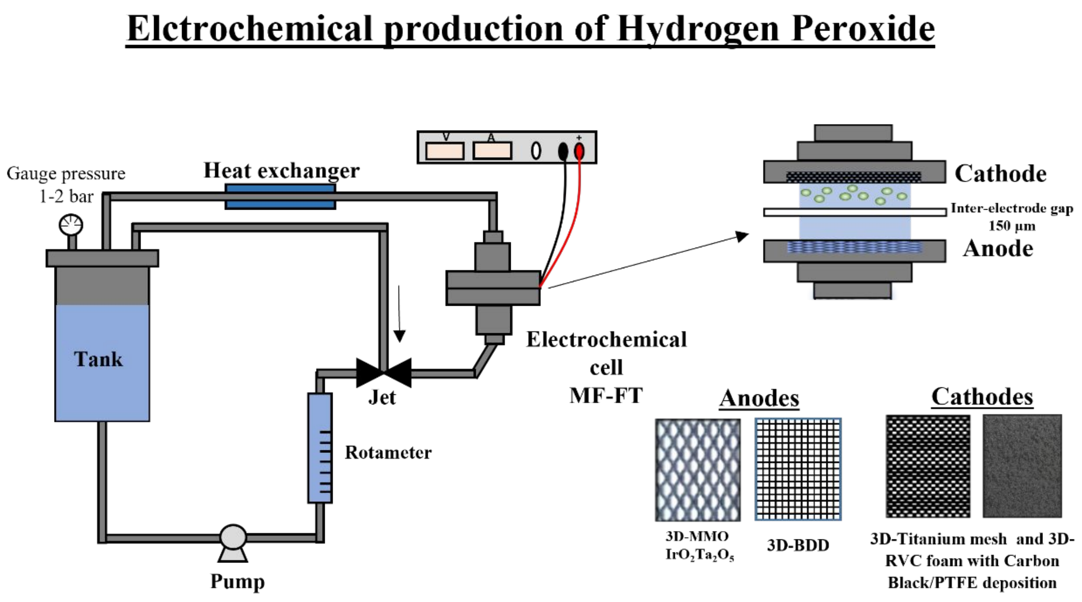


**Figure S1.** Schematic representation of the experimental set-up for the electrochemical production of hydrogen peroxide.

# Production of chlorine dioxide

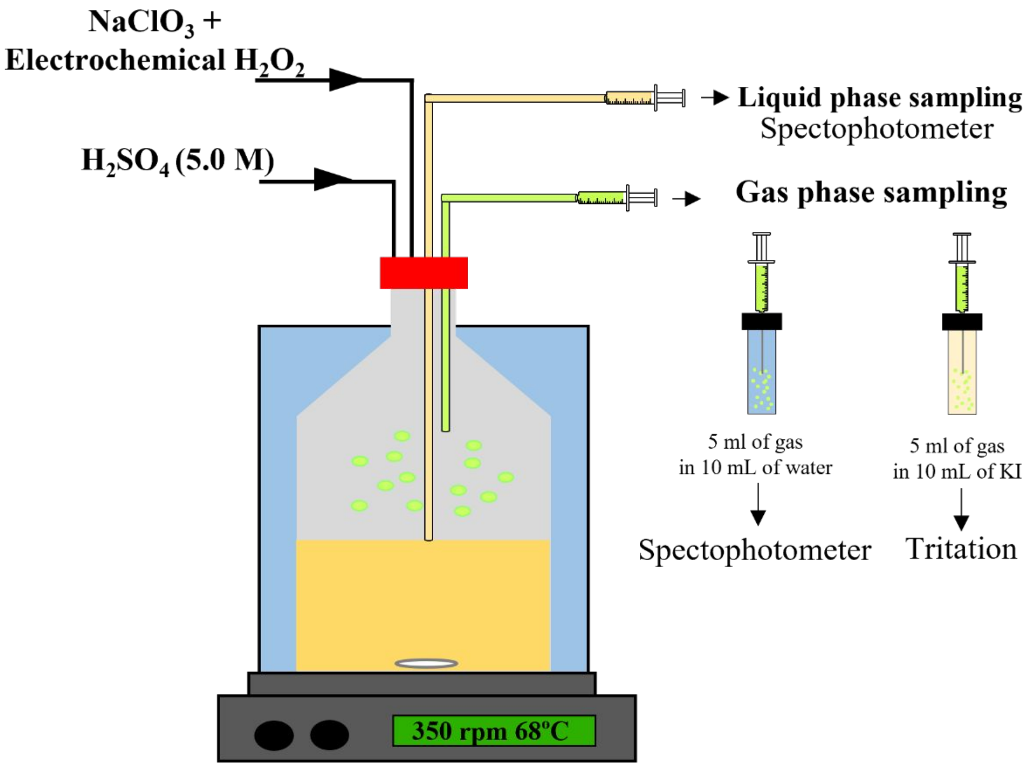


**Figure S2.** Schematic representation of the experimental set-up for the production of chlorine dioxide.
